# Metabolic reprogramming in esophageal squamous cell carcinoma

**DOI:** 10.3389/fphar.2024.1423629

**Published:** 2024-06-26

**Authors:** Ziyi Wang, Xiangyu Sun, Zehui Li, Huidong Yu, Wenya Li, Yan Xu

**Affiliations:** ^1^ Department of Surgical Oncology and General Surgery, First Hospital of China Medical University, Shenyang, Liaoning, China; ^2^ Department of Thoracic Surgery, National Cancer Center/National Clinical Research Center for Cancer/Cancer Hospital, Chinese Academy of Medical Sciences and Peking Union Medical College, Beijing, China; ^3^ Department of Thoracic Surgery, First Hospital of China Medical University, Shenyang, Liaoning, China; ^4^ Department of Breast Surgery, Liaoning Cancer Hospital and Institute, Cancer Hospital of Dalian University of Technology, Cancer Hospital of China Medical University, Shenyang, Liaoning, China

**Keywords:** metabolic reprogramming, glucose, fatty acid, amino acid, esophageal cancer

## Abstract

Esophageal squamous cell carcinoma (ESCC) is a malignancy with high incidence in China. Due to the lack of effective molecular targets, the prognosis of ESCC patients is poor. It is urgent to explore the pathogenesis of ESCC to identify promising therapeutic targets. Metabolic reprogramming is an emerging hallmark of ESCC, providing a novel perspective for revealing the biological features of ESCC. In the hypoxic and nutrient-limited tumor microenvironment, ESCC cells have to reprogram their metabolic phenotypes to fulfill the demands of bioenergetics, biosynthesis and redox homostasis of ESCC cells. In this review, we summarized the metabolic reprogramming of ESCC cells that involves glucose metabolism, lipid metabolism, and amino acid metabolism and explore how reprogrammed metabolism provokes novel opportunities for biomarkers and potential therapeutic targets of ESCC.

## Introduction

Esophageal cancer is a common malignancy of the digestive tract. The new cases of esophageal cancer have exceeded 6,00,000 annually, ranking the seventh among all cancers. The death cases of esophageal cancer are about 5,40,000 annually, ranking the sixth among all cancer types (Sung et al., 2021). Esophageal squamous cell carcinoma (ESCC) and esophageal adenocarcinoma (EAC) are the two main histological subtypes of esophageal cancer (Rogers et al., 2022). As a high-risk country of esophageal cancer, more than 90% of the cases diagnosed in China are ESCC (Liu CC et al., 2019). With the improvement of early diagnosis and comprehensive therapeutic strategies for ESCC, the therapeutic effect of esophageal cancer has been improved, but the 5-year overall survival rate of ESCC is only 15%∼20% (Huang and Yu, 2018; Zhu et al., 2023).

Metabolic reprogramming is an emerging hallmark of tumors (Kroemer and Pouyssegur, 2008). To adapt to hypoxia and nutrient deprivation, proliferating tumor cells need to reprogram metabolic pathways to fulfill the increasing demands of energetic production, cellular building components and redox balance of tumor cells (Pavlova and Thompson, 2016). A series of genetic alteration and cellular signaling are involved in the regulation of diverse aspects of cellular metabolism (DeBerardinis and Chandel, 2016). Studies have identified profound metabolic reprogramming in aggressive ESCC that involves glycolysis, tricarboxylic acid (TCA) cycle, lipid metabolism, and glutamine addiction, etc. Spatially resolved metabolomics in 256 ESCC patients revealed that multiple altered metabolic pathways, including proline biosynthesis, glutamine metabolism, uridine metabolism, histidine metabolism, fatty acid biosynthesis and polyamine biosynthesis participate in the pathogenesis of ESCC. Notably, metabolic enzymes pyrroline-5-carboxylate reductase 2 and uridine phosphorylase 1 are therefore identified as potential therapeutic targets. Huang et al. (2024) integrated the single cell RNA-seq with metabolomics of ESCC tissues and plasma samples, indicating that nicotinate and nicotinamide metabolism pathway was dysregulated in ESCC patients with lymphatic metastasis with significant 1-methylnicotinamide upregulation. In this review, we primarily discussed the advantages of metabolic reprogramming for ESCC cells and explored how reprogrammed metabolism provokes novel opportunities for biomarkers and potential therapeutic targets of ESCC.

## Reprogrammed metabolic pathways in ESCC

### Glucose metabolism and ESCC

**FIGURE 1:**
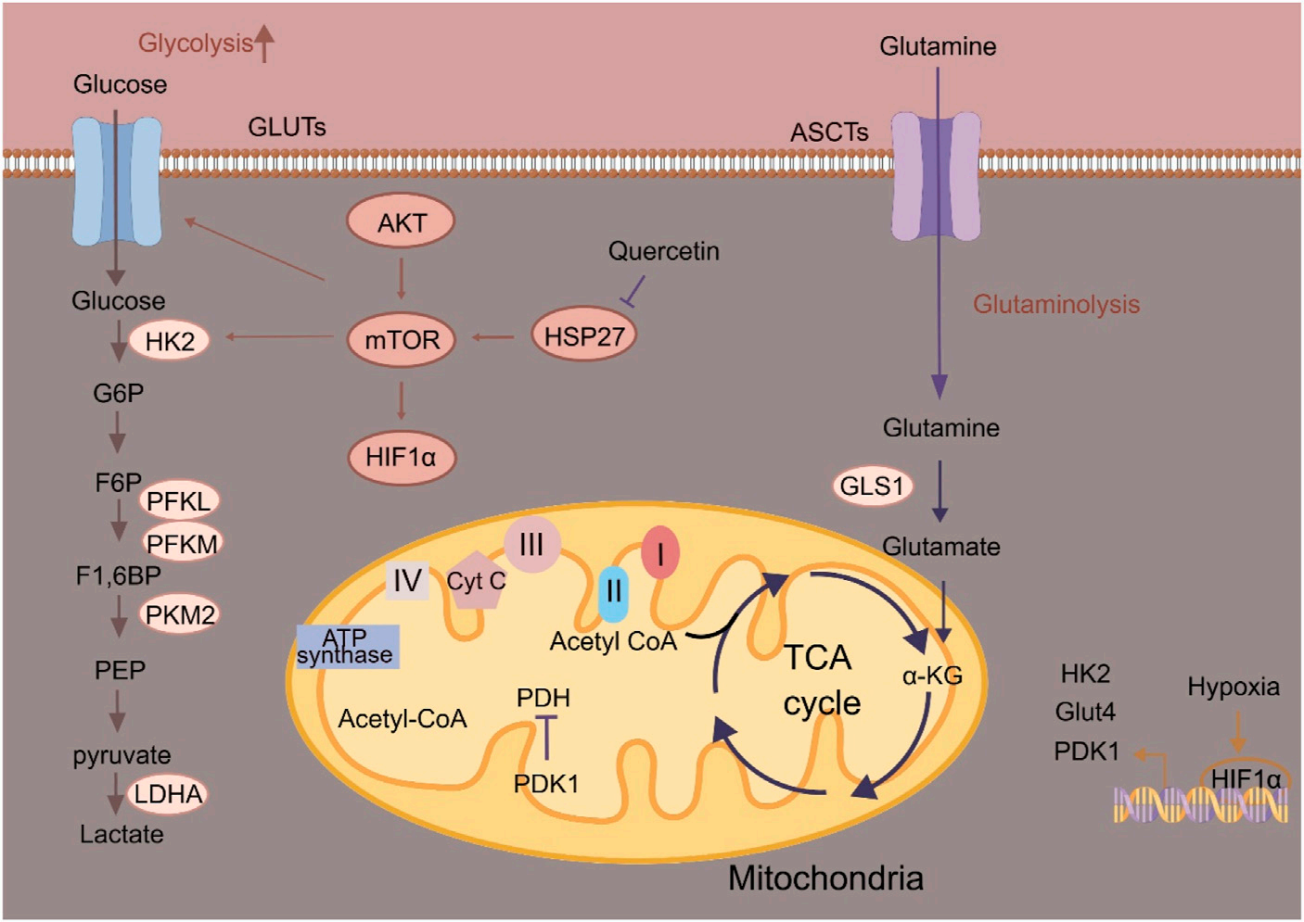
Schematic illustration of glucose metabolism in ESCC cells. α-KG, α-ketoglutarate; ASCT, Alanine serine cysteine transporter; Cyt C, Cytochrome c; F-1, 6-BP, Fructose-1, 6-bisphosphatase; G6P, Glucose-6-phosphate; Glut, Glucose transporter; GLS1, Glutaminase 1; HK2, Hexokinase 2; HIF-1α, Hypoxia-inducible factor 1α; HSP27, Heat shock protein 27; LDHA, Lactate dehydrogenase A; PDK1, 3-phosphoinositide-dependent kinase 1; PFK, Phosphofructokinase; PKM2, Pyruvate kinase M2.

Altered glucose metabolism is commonly observed in ESCC cells (Figure 1). Like other tumors, glycolysis is the preferred energetic pathway in ESCC cells (Liu et al., 2021; Su et al., 2022). Depending on glycolysis, ESCC cells convert glucose to lactate to rapidly produce ATP and promote tumor progression (Wang et al., 2021; Li et al., 2023). The glycolytic pathway plays an essential role in ESCC metabolism, as shown by the deregulation of multiple metabolic enzymes. As the first rate-limiting enzyme in glycolysis, hexokinases (HKs) catalyze the phosphorylation of glucose to glucose-6-phosphate. Among the four isoforms, HK2 exerts greater effect in promoting aerobic glycolysis than other isoforms (Ciscato F et al., 2021). Liu K et al. (2019) found that HK2 is essential for maintaining esophageal cancer stem cell (CSC) phenotypes. Esophageal CSCs exhibit higher glycolysis, which are regulated via the small heat shock protein 27 (Hsp27)-AKT-HK2 axis. Hsp27 could increase the expression of HK2 through activating AKT-mTOR in esophageal CSCs. Quercetin, the Hsp27 inhibitor, could augment the anti-ESCC effects of adjuvant chemotherapy. Phosphofructokinases (PFKs) catalyze the phosphorylation of fructose-6-phosphate to fructose-1, 6-bisphosphate, and has three PFK isozymes including liver type (PFKL), platelet type (PFKP) and muscle type (PFKM) (Feng et al., 2020). PFKL overexpression is associated with reduced survival of ESCC patients, and its inhibition suppresses ESCC growth (Zheng et al., 2022). Antipsychotic drug penfluridol directly targets PFKL to impair glycolytic pathway and only exhibits its anti-tumor effect in PFKL-proficient tumors (Zheng et al., 2022). Under hypoxia, GHRH-R splice variant 1 (SV1) is elevated to upregulate PFKM to promote glycolytic pathway and enhance development of ESCC (Gu et al., 2022). MIA-602 (a SV1 inhibitor) could block the oncogenic role of PFKM in ESCC. 6-phosphofructo-2-kinase/fructose-2, 6-biphosphatase 3 (PFKFB3) mediates the synthesis and degradation of fructose 2, 6-bisphosphate. PFKFB3 could increase glycolytic flux in ESCC cells, as well as ESCC cell cycle progression and growth. lncRNA Actin Gamma 1 Pseudogene (AGPG) has been identified to bind to and stabilize PFKFB3 by preventing APC/C-mediated ubiquitination of PFKFB3 and stabilizing PFKFB3 protein (Liu et al., 2020). Pyruvate kinases (PKs), the last rate-limiting enzymes in glycolysis, catalyze the conversion of phosphoenolpyruvate to pyruvate. There are four isoforms of PKs, including PKL (found in liver), PKR (red blood cell), PKM1 and PKM2 (found in muscle) (Israelsen and Vander Heiden, 2015). The critical role of glycolysis in ESCC development is further supported by the correlation between increased PKM2 expression and poor prognosis. PKM2 has been found to be upregulated in ESCC patients (Ma et al., 2019). circCYP24A1 interacts with PKM2 to enhance C-C chemokine ligand 5 (CCL5) production and development of ESCC (Gu et al., 2022). Methyltransferase-like 3 induces the m6A modification of APC by recruiting YTH domain family (YTHDF) for APC mRNA degradation, which further upregulates β-catenin and its downstream targets PKM2 to enhance aerobic glycolysis and promote ESCC progression. PKM2 has also been found to be a target of photodynamic therapy (PDT) (Li B et al., 2021). PDT inhibits PKM2 and activates caspase-3/8 to release Gasdermin E-N (N-GSDME) and induce pyroptosis in ESCC cells. Lysyl-oxidase like-2 (LOXL2) and its catalytically inactive L2Δ13 splice variant interact physically with aldolase A, glyceraldehyde-3-phosphate dehydrogenase and enolase to boost glycolysis of ESCC cells, therefore promoting ESCC progression (Jiao et al., 2022). Pyruvate dehydrogenase kinase 1 (PDK1) is a Ser/Thr kinase that inactivates mitochondrial pyruvate dehydrogenase, leading to a metabolic reprogramming from oxidative phosphorylation (OXPHOS) to glycolytic pathway. PDK1 is significantly overexpressed in ESCC tissues and cell lines compared with the normal tissues or cells (Ma et al., 2023). PDK1 could suppress cell proliferation of ESCC by blocking glycolytic pathway (Ma et al., 2023). Hypoxia-inducible factor 1α (HIF-1α) is the key transcriptional regulator of glycolysis, mediating cellular adaptation to hypoxia. In ESCC, long intergenic noncoding RNA for kinase activation (LINK-A) disrupts the interaction between MCM3 and HIF-1α, reducing MCM3-mediated HIF-1α transcriptional inhibition to enhance glycolytic phenotype and chemoresistance. m6A demethylase fat mass and obesity-associated protein (FTO) could stabilize LINK-A, making the FTO/LINK-A/MCM3/HIF-1α axis as a potential target for anti-ESCC strategy (Nan et al., 2023). LncRNA MALR upregulation leads to ILF3 liquid-liquid phase separation and activates HIF1α to promote ESCC development (Liu et al., 2023). Hypoxia-induced lncRNA G077640 upregulation promotes ESCC progression by stabilizing HIF1α to upregulate glucose transporter 4 (GLUT4), HK2 and PDK1 (Huang et al., 2023). Targeting glycolytic rate-limiting enzymes, glucose transporters and other metabolic enzymes are considered targets for screening potential anti-tumor drugs. However, based on *in vitro* or *in vivo* experiments, most anti-ESCC drugs that target glucose metabolism are still in the development stage.

The TCA cycle is indispensable for OXPHOS to fulfill the needs of bioenergetics and biosynthesis (Nolfi-Donegan et al., 2020). The regulation of mitochondrial biogenesis is essential for sustaining redox homeostasis to boost anti-immunity and inhibit tumor progression (Zong et al., 2016). Mitochondrial transcription factor A (TFAM) has been found to be downregulated in ESCC, and TFAM downregulation is correlated with poor survival of ESCC patients. TFAM is essential for replication and stability of mitochondrial genome, and TFAM inhibition induces mitochondrial DNA (mtDNA) release into the cytosol and activates the cGAS-STING signaling to induce autophagy and ESCC proliferation. Further, STING abrogation or mtDNA degradation by DNase I impairs TFAM-depleted ESCC cell proliferation (Li et al., 2022). CircPUM1 is positively associated with the expression of HIF1α under hypoxia in ESCC cells. CircPUM1 regulates the mitochondrial complex III assembly through interacting with mitochondrial ubiquinol-cytochrome c reductase core protein (UQCRC), which ultimately promotes OXPHOS for ATP production to facilitate proliferation and inhibit pyroptosis of ESCC cells (Gong et al., 2022). In ESCC, oncogenic STAT3 signaling pathway participates in regulating the activity of electron transport chain (ETC). STAT3β could inhibit the phosphorylation of STAT3α at S727 in mitochondria via ERK1/2 to impair the activity of ETC, therefore activating GSDME for pyroptosis and sensitizing ESCC cells to cisplatin (Zheng et al., 2021). Targeting OXPHOS in ESCC cells as an anti-ESCC strategy deserves further investigations.

The pentose phosphate pathway is a branch from glycolysis and serves as a major source of ribonucleotides and NADPH. In ESCC, downregulation of polo like kinase 1 (PLK1) could block the pentose phosphate pathway to impair the production of NADPH, thereby enhancing ferroptosis and promoting the sensitivity of ESCC cells to chemotherapy (Zhao et al., 2023).

### Lipid metabolism and ESCC

**FIGURE 2:**
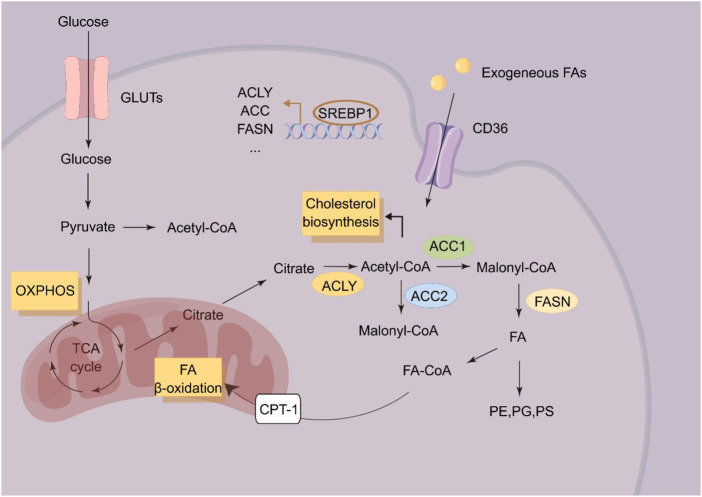
Schematic illustration of lipid metabolism in ESCC cells. ACC, acetyl-CoA; ACLY, ATP citrate lyase carboxylase; CPT-1, Carnitine palmitoyl transferase 1; FA, Fatty acid; FASN, Fatty acid synthase; PE, Phosphatidylethanolamine; PS, Phosphatidylserine; PG, Phosphatidylglycerol; SREBP1, Sterol response element binding protein-1.

Altered lipid metabolism is a prominent metabolic reprogramming in ESCC (Figure 2). Enhanced lipid biosynthesis or uptake is required for ESCC progression (Cheng et al., 2018). Fatty acid synthesis is a process that begins with the carboxylation of acetyl-CoA to malonyl-CoA by acetyl-CoA carboxylase. Malonyl-CoA is further committed to fatty acid synthesis by producing mainly 16-C palmitate via fatty acid synthase (FASN). FASN has been found to promote the progression of ESCC. circHIPK3 acts as a ceRNA by sponging miR-637 to upregulate FASN expression and fatty acid biosynthesis of ESCC cells, indicating circHIPK3/miR-637/FASN axis as a promising therapeutic target for anti-ESCC strategy (Cao et al., 2024). *De novo* lipogenesis is transcriptionally regulated by sterol regulatory element binding protein (SREBP) (Eberlé et al., 2004). Overexpression of SREBP1 is correlated with poor prognosis in ESCC patients, and supports ESCC progression by enhancing fatty acid biosynthesis. In ESCC, pre-mRNA processing factor 19 (PRP19) enhances the stability of SREBP1 mRNA in an N6-methyladenosine-dependent manner to mediate SREBP-dependent fatty acid synthesis and ESCC progression. SREBP could also cooperate with TP63/Kruppel like factor 5 (KLF5) to regulate the biosynthesis of fatty acids (Zhang et al., 2023).

Fatty acid oxidation is the process involving shortening of fatty acids and producing in acetyl-CoA, NADH and FADH2 (Ma et al., 2018). Carnitine O-palmitoyl transferase 1 (CPT1A), the key rate-limiting enzyme in fatty acid oxidation, is upregulated in ESCC, which is correlated with poor survival of ESCC patients (Tian et al., 2022). In ESCC, CPT1A could inhibit cellular apoptosis by providing GSH and NADPH to sustain redox homeostasis. Genetic or pharmacologic inhibition of CPT1A reduces the NADPH supply to impair anchorage-independent growth of ESCC cells *in vitro* and *in vivo* (Tian et al., 2022).

Cholesterol is an important component of cell and organelle membranes and a precursor of steroid hormones and bile acids (Huang et al., 2020). The rapid proliferation of tumor cells requires enhanced biosynthesis of cell and organelle membranes, and aberrant cholesterol metabolism therefore plays a pivotal role in tumor initiation and progression (Xu et al., 2020). Metastatic ESCC cells display elevated cholesterol accumulation via upregulating the expression of anoctamin 1 (ANO1). ANO1 results in cholesterol accumulation by blocking LXR signaling and inhibiting cholesterol hydroxylation by downregulating hydroxylase CYP27A1 (Singh and Mehla, 2023). Lysophosphatidylcholine acyltransferase 1 (LPCAT1) has been found to be upregulated in ESCC tissues, and LPCAT1 could rewire cholesterol metabolism of ESCC cells. LPCAT1 could upregulate the expression of SQLE to promoting the entry of SP1/SREBP2 into the nucleus via activating PI3K. LPCAT1 also promotes the entry of SREBP1 into the nucleus by EGFR-mediated INSIG-1 downregulation, leading to enhance cholesterol biosynthesis (Tao et al., 2021).

Phospholipid is composed of phosphoglyceride and sphingomyelin. Phosphoglyceride includes phosphatidylcholine, phosphatidylserine, phosphatidylglycerol, diphosphatidylglycerol, phosphatidylethanolamine, and phosphatidylinositol. In ESCC, DNA hypermethylation of otubain 2 (OTUB2) has been found to induce tumor initiation and chemoresistance by regulating biosynthesis of phosphatidylserine. Mechanistically, OTUB2 mediates the deubiquitination and phosphorylation of STAT1 and further promotes calmodulin-like protein 3 (CALML3) upregulation. Subsequently, CALML3 regulates mitochondrial calcium signaling to promote OXPHOS and biosynthesis of phosphatidylserine. More importantly, orally-administered phosphatidylserine impairs tumor initiation of OTUB2-low ESCC cells in mouse models, making phosphatidylserine administration as a potential anti-ESCC strategy (Chang et al., 2022). Fatty acid 2-hydroxylase (FA2H), catalyzing the hydroxylation of fatty acids, has been found to be significantly enriched in a subpopulation of ESCC cells with high metastatic potential, and that FA2H inhibition significantly blocks metastatic potential of ESCC cells. Notably, Ceramide (d18:0/24:0) and Ceramide (d18:0/24:1) are increased in FA2H-knockdown ESCC cells. Upon administration, Cer (d18:0/24:0) and Cer (d18:0/24:1) significantly blocks tumor metastasis in mouse models (Zhou et al., 2022).

Currently, multiple studies have revealed that lipid metabolism in ESCC tissues is significantly altered compared with normal esophageal tissues, which may lead to the occurrence and development of ESCC. Therefore, targeting lipid metabolism as a potential therapeutic approach will provide new opportunities for the treatment of ESCC. However, there are still challenges to overcome in techniques of lipid detection. The accuracy of mass spectrometry-based lipidomics is required to be improved for more accurate qualitative and quantitative analysis. The combined analysis of multi-omics could reveal the biological feature of the occurrence and development of ESCC comprehensively. The combination of lipidomics with genomics, transcriptomics and proteomics may provide more insights for the treatment ESCC.

### Amino acid metabolism and ESCC

Cancer cells exhibit elevated demand for amino acids to support their fast proliferation (Sivanand and Vander Heiden, 2020). It has been found that tumor cells exhibit greater dependence on glutamine (Cluntun et al., 2017). Glutamine participates in cellular bioenergetics through α-ketoglutarate in TCA cycle and serves as a major source of nitrogen for nucleotide biosynthesis. In ESCC, genetic alterations including Fbxo4 loss and hyperactivation of cyclin D1-CDK4/6 kinases have been frequently observed, leading to glutamine addiction (Qie et al., 2019). The Fbxo4-cyclin D1 axis could regulate glutamine consumption and mitochondrial dysfunction by suppressing Rb activity and activating mTORC1 to promote ESCC development. Glutaminase 1 (GLS1) is a rate-limiting glutaminolysis enzyme that transforms glutamine into glutamate to support proliferation of ESCC cells. Combined treatment of CB-839 (GLS1 inhibitor) and metformin could synergistically impair proliferation of ESCC cells, providing promising therapeutic strategy for anti-ESCC treatment. Glutamine metabolism is also regulated by RNA binding motif protein 4 (RBM4)-LKB1 axis to enhance ESCC cell survival. RBM4 binds with LKB1 to impair the LKB1/STRAD/MO25 complex and promote TRIM26-mediated LKB1 ubiquitination and degradation to overcome cell senescence (Chen et al., 2023). NEDD4 like E3 ubiquitin protein ligase (NEDD4L) blocks glutamine metabolism in ESCC by ubiquitination of c-Myc to downregulate GLS1 and SLC1A5, which suppresses tumor progression (Cheng et al., 2022). Collectively, targeting glutamine metabolism in cancer therapy brings new opportunities for anti-ESCC strategy.

Arginine functions as the precursor for synthesis of protein, nitric oxide, polyamines, agmatine, creatine, and urea (Chen et al., 2021). Of the enzymes catalyzing rate-controlling steps in arginine metabolism, argininosuccinate synthetase 1 (ASS1) and argininosuccinate lyase (ASL) levels were increased in ESCC tissues, but reduced in metastatic ESCC tissues (Sun et al., 2024). Blocking ASS1 or ASL could impair ESCC growth at the primary site and promote distant metastasis (Sun et al., 2024). The gene encoding 26S proteasome non-ATPase regulatory subunit 2 (PSMD2) activates the mTOR pathway by upregulating ASS1 to inhibit autophagy and promote ESCC progression (Liu et al., 2023). Under hypoxia, receptor tyrosine kinase IGF1R is upregulated in ESCC to elevate the transcription of ASS1 through c-MYC to reprogram arginine metabolism (Fang et al., 2023).

Although nutritional epidemiological study has shown that high methionine intake is not associated with ESCC risk, Jin et al. (2024) found that methionine cycling has been hyperactivated in ESCC tissues and correlated with poor survival outcome. ESCC cells prefers to utilize exogenous methionine to produce S-adenosine methionine (SAM), leading to enhanced ESCC cell proliferation. Mechanistically, methionine enhances METTL3-mediated RNA m6A methylation through SAM and upregulates NR4A2 expression. Celecoxib has been identified as a potent NR4A2 inhibitor with promising anti-ESCC potential.

More comprehensive analysis of amino acid metabolic reprogramming and its related metabolic pathways in ESCC cells should be conducted to assist developing more effective anti-ESCC strategies. However, there are still questions waiting for answer. For example, whether metabolic reprogramming in amino acid cooperates with other related pathways to sustain tumor development? How other metabolic pathways compensate to promote ESCC cell proliferation once amino acid metabolism is dysregulated in ESCC cells? What is the therapeutic effect of the usage of specific amino acid-limited diet to treat ESCC?

## Clinical implications of metabolism-targeted therapy in ESCC

Metabolomics cooperates with genomics, transcriptomics and proteomics to form “systems biology,” which plays an important role in cancer research. Metabolomics mainly includes targeted metabolomics and non-targeted metabolomics. Untargeted metabolomics works by detecting all metabolites in the samples to obtain quantitative information to decipher the differences in metabolites between groups. Targeted metabolomics analyzes a limited number of metabolites associated with biological process (Schrimpe-Rutledge et al., 2016). Based on the tissue metabolic profiles consistently identified by nuclear magnetic resonance and targeted mass spectrometry techniques, Zhao et al. (2024) developed nuclear magnetic resonance -based serum and urine metabolic profiles and optimized to reliably reflect the metabolic profiles of ESCC. Due to the metabolic adaptability during tumor progression and treatment, it is important to track and monitor metabolic adaptability of ESCC cells during tumor progression and treatment. Therefore, it is necessary to dynamically and accurately observe metabolic alterations and change treatment strategies accordingly. Optimization of existing detection methods and searching for alternative non-invasive detection methods are key research directions (Xiao et al., 2023).

One strategy for discovering anti-ESCC therapy is to screen FDA-approved drugs. Recent study reported that antitussive agent cloperastine inhibits the proliferation of ESCC *in vivo* and *in vitro* by impairing mitochondrial OXPHOS (Li L et al., 2021; Li LY et al., 2021). Liu Y et al. (2023) found that sulconazole can effectively block the growth of ESCC cells by inducing mitochondrial oxidative stress and inhibiting glycolysis. The current reported metabolism-targeted therapy has been summarized in Table 1. Metabolic enzymes are attractive therapeutic targets in anti-tumor therapy, but new drugs targeting metabolism have been limited due to toxicity to normal tissues. And more studies have recognized that some metabolic enzymes drive tumor progression through non-catalytic mechanisms (Pan et al., 2021). Preclinical studies have identified several metabolic molecules that inhibit tumor progression as potential therapeutic targets, and some are already in clinical trials (Xiao et al., 2023). However, at present, metabolic therapy for ESCC patients is still stuck in pre-clinical research, and it is urgent to further explore new targets and optimal treatment strategies. The goal of identifying and tracking metabolic targets is to enable precision therapy. Considering the heterogeneity of metabolism in tumors, individualized metabolism-targeted therapy will be the future development direction.

**TABLE 1:** Targeting cellular metabolism for potential anti-ESCC strategy.

Drug	Target	Metabolic effects	Anti-tumor effects	Reference
Targeting glucose metabolism				
Quercetin	Hsp27	Suppressing Hsp27-AKT-HK2 axis to block glycolysis	Increasing sensitivity to chemotherapy	Liu CC et al. (2019)
Penfluridol	PFKL	Suppressing glycolysis	Inhibiting proliferation *in vitro* and growth *in vivo*	Zheng et al. (2022)
MIA-602	SV1	Suppressing SV1-PFKM axis to block glycolysis	Inhibiting cell cycle progression and growth	Gu et al. (2022)
Cloperastine	—	Suppressing OXPHOS	Inhibiting proliferation *in vitro* and growth *in vivo*	Li L et al. (2021)
DNase I	mtDNA	Suppressing OXPHOS	Inhibiting TFAM-depleted ESCC cell proliferation	Li et al. (2022)
Sulconazole	—	Suppressing glycolysis, inducing oxidative stress	Inducing PANoptosis, increasing radiosensitivity Inhibiting cell proliferation and migration	Liu et al. (2023)
Targeting lipid metabolism				
Perhexiline	CPT1A	Suppressing fatty acid oxidation	Inhibiting the anchorage-independent growth of ESCC cells *in vitro* and lung metastases of xenografted tumor models *in vivo*	Tian et al. (2022)
Phosphatidylserine	OTUB2-low	DNA hypermethylation of OTUB2 promotes OXPHOS and biosynthesis of phosphatidylserine	Impairing tumor initiation and chemoresistance of OTUB2-low ESCC cells in mouse models	Chang et al. (2022)
Ceramide (d18:0/24:0) and Ceramide (d18:0/24:1)	FA2H-low	—	Blocking metastatic potential of ESCC *in vitro* and vivo	Zhou et al. (2022)
Targeting amino acid metabolism				
CB-839	GLS1	Suppressing glutamine addiction	Combined with metformin to overcome acquired resistance to CDK4/6 inhibitors *in vitro* and *in vivo*	Qie et al. (2019)
	GLS1	Suppressing glutamine addiction	Inducing cellular senescence and sustaining cell proliferation	Chen et al. (2023)

Collectively, exploring the anti-ESCC strategy in the context of tumor metabolism has unveiled novel and promising opportunities, as well as more comprehensive understandings of metabolic reprogramming of ESCC cells. In the future, more knowledge of the metabolic reprogramming of ESCC cells are required to refine rational anti-ESCC strategies that target tumor metabolism.
